# Obesity in the Pediatric Population of the National (Nationwide) Inpatient Sample (NIS), USA

**DOI:** 10.7759/cureus.33111

**Published:** 2022-12-29

**Authors:** Okelue E Okobi, Ijeoma C Izundu, Endurance O Evbayekha, Emmanuel O Egberuare, Esther O Segun, Rafiat A Abdulgaffar, Babatunde O Oyelade, Jenny J Onyema, Tariladei S Peresuodei, Scholastica Uyileubenye Abu-Undiyaundeye

**Affiliations:** 1 Family Medicine, Arizona State University, Tempe, USA; 2 Family Medicine, Lakeside Medical Center, Belle Glade, USA; 3 Pediatrics and Child Health, iheed, Royal College of Physicians of Ireland (RCPI), Dublin, IRL; 4 Post-Anaesthetic Care Unit, Markham Stouffville Hospital, Markham, CAN; 5 Internal Medicine, St. Luke's Hospital, Chesterfield, USA; 6 Urology, Prostate Cancer Center, Calgary, CAN; 7 School of Biological Sciences & Applied Chemistry, Seneca College, Toronto , CAN; 8 Communicable Disease Control, Alberta Health Services, Calgary, Calgary, CAN; 9 Cardiology, Emory University, Atlanta , USA; 10 Internal Medicine, Igbinedion University, Benin, NGA; 11 Medicine, Kent and Canterbury Hospital, Canterbury, GBR; 12 Family Medicine, Madonna University, Elele, NGA

**Keywords:** overweight and obesity, obesity, community obesity, childhood overweight, “childhood obesity morbidity”

## Abstract

Background: The incidence of childhood obesity has received a lot of attention lately, especially in the United States. The increased prevalence of pediatric obesity and its association with comorbidities has piqued the attention of more scientists in the epidemic's patterns. Our research examined the National (Nationwide) Inpatient Sample (NIS) data set for hospitalized persons aged 18 years or younger with primary or secondary obesity between 2016 and 2019 to investigate the prevalence, risk factors, and related diseases.

Methods: We retrospectively examined individuals with primary or secondary obesity from 2016 to 2019 using the NIS database. To extract the weighted samples, we utilized the International Classification of Diseases (ICD)-10 diagnostic codes E66, E660, E6601, E6609, E662, E668, and E669. Individuals with drug-related obesity or obesity caused by a recognized pathologic disease unrelated to high-calorie intake were excluded. First, we queried the total population, then separated them by age category and picked our population of interest, i.e., those aged 18 and under. The NIS is a deidentified database available to the public. It collects data on around 8 million hospitalizations annually, accounting for roughly 20% of all admissions in the United States.

Results: The findings show that between 2016 and 2019, prevalence rates of childhood obesity were still on the rise and plateaued in 2019. There were 28,484,087 study subjects in this weighted sample between 2016 and 2019. Of these, 13.9% (3,946,889) were diagnosed with obesity. The sample population for those 18 years of age or under was 62,669 (1.5%) children with obesity with a mean age of 14 (SD = 4). Also, there was a 64.2% female preponderance. The obtained yearly showed a steady and significant rise from 2016 to 2018 (24% vs. 26%), with a slight decline in 2019 (25%; p < 0.001). Even though the white population had the highest overall prevalence of childhood obesity (40.9%), the Hispanic and black people had a higher prevalence per population, with a 0.5% and 0.33% prevalence, respectively, compared to 0.14% in the white population (p < 0.0001). When geographical regions were considered, south had the highest rate (36.40%), followed by the west (24.71%) and the midwest (23.56%). The analysis also showed that people with lower median household income (0-25th percentile) had the highest rate of childhood obesity (38.17%) compared to higher-income earners (13.19%).

Conclusion: In our finding, obesity in the pediatric population is still increasing, continuing on its previously recorded trajectory. Various recommendations from health policymakers have bolstered efforts to tackle this escalating pandemic. However, additional information on the compliance, use, and adherence to these policies by healthcare professionals and members of the public, as well as the consequence of utilization or compliance to these guidelines, is needed. Nevertheless, given the continuous growth of childhood obesity, despite the avalanche of these recommendations, the issue of compliance arises, or other essential risk factors might have been overlooked. Additional studies may be needed to unmask this looming phenomenon.

## Introduction

Being overweight and obese, in general, has been a significant health risk factor leading to many other diseases. Its incidence and prevalence are soaring high globally [1-5]. But childhood obesity has been generally overlooked, underdiagnosed, and undertreated in various societies because of factors like illiteracy, ignorance, underrecognition by health workers, or societal belief that a "fat child" is synonymous with a healthy child [1-3]. These subtle but dangerous physical characteristics in some children triggered the presentation to be classified as a global public health epidemic. Obesity is generally known, and excess accumulation of body fat and the body mass index (BMI) is the most used global indices to classify individuals into underweight, normal weight, overweight, or obese in the adult population [2].

In contrast to the uniformity of calculating a person's weight in kilograms by the square of their height in meters for BMI measurements in the adult population, BMI in the pediatric population is sex- and age-specific. As a result, BMI in children is frequently stated compared to their peers of the same age and gender [1]. Therefore, the definition of childhood overweight and obesity varies amongst health policy-making and regulatory authorities. Efforts have been made to unify these pockets of varying definitions to have a unified front in establishing a benchmark to fight this epidemic. The challenges in uniformly defining overweight and obesity in the pediatric population using a single index of measurement stems from various factors, including accuracy of measurement and calibration tools, the rapidly ever-changing physiologic growth factors that happen in this unique subset of the population, and their variations in diverse ethnic backgrounds, sex and age groups [4-5]. For example, a three-year-old male child from Chinese parents may significantly vary in weight and length than that of a baby girl of German or American descent because of underlying genetics, sex, hormonal environment, and other interplays that drive their growth rates in children. However, the pediatric population generally varies per age bracket (preschool, school-aged children, and adolescents) compared to overweight and obesity in adults, which have unified standard benchmarks for defining them. We shall review definitions, implications, and guidelines for managing this health epidemic by focusing on the WHO, the American Academy of Pediatrics, the Center for Disease Control, and the Canadian Pediatric Society.

The WHO, to describe the enormity of this condition that affects all socioeconomic groups and ages and the rate at which its aftermath causes health disorders globally, and paradoxically occurring even in areas of undernutrition, termed it "globesity." In 2006, it defined overweight and obesity in various ways using the WHO child growth standard chart for children aged 0-5 years, classified into weight-for-length/height and BMI-for-age and corrected for age and sex. Then in 2007, using the WHO Growth Reference charts for children aged 5-19 years, they defined overweight as equal to one standard deviation BMI for age and sex, and obesity as two standard deviations BMI for age and sex for children [2]

Similarly, in Canada, in 2010, with mounting evidence indicating the development trends of well-fed, healthy preschool children from various ethnic origins, a joint statement from the College of Family Physicians of Canada, Canadian Pediatric Society, Dietitians of Canada, and Community Health Nurses of Canada embraced the WHO growth charts in an effort to promote consistent growth monitoring and assessment practices for weight gain and linear growth in the pediatric population [4]. They re-echoed using the WHO's growth standard charts for all full-term to age five children and the WHO growth reference 2007 charts for ages 5-19. They also recommended that for all children two years of age and older, the BMI for the period should be used to measure weight relative to height and to screen for thinness, wasting, overweight, and obesity. For youngsters under two, weight for length or body weight percentile may be employed [4]. They then recommended that these measures be ranked in centiles, with specific cut-off values to be used as an indication for referral for intervention rather than diagnostic criteria. In line with this, they defined overweight and obesity in 0-2 years as greater than the 97th percentile and 99.9th percentile of weight for length, respectively. In addition, in children between 2-5 years, they classified this condition, using BMI-for-age, into overweight (> 97th centile) and obesity (> 99.9th centile). For kids between 5-19 years, this excess body fat condition was further subclassified into overweight (> 85th percentile), obesity (> 97th percentile), and severe obesity (> 99.9th percentile) [4].

In the United States, the Centers for Disease Control and Prevention (CDC) growth charts are routinely used to assess children's size and growth trends. This CDC growth chart incorporates BMI-for-age since BMI is gender- and age-specific in children and is expressed in percentiles when adjusted for gender and age. In addition, the expert committee advised that overweight be defined as the 85th to less than the 95th percentile and obesity as higher than the 95th percentile, equivalent to the threshold for developing a poor health outcome linked with obesity [1,2,5].

The International Obesity Task Force (IOTF) is another well-respected health organization that has attempted to define obesity. In an international survey of six prominent nationally representative cross-sectional growth studies in Hong Kong, Brazil, the Netherlands, the United Kingdom, the United States, and Singapore, the BMI of approximately 97,876 males and 94,851 females from birth to 25 years of age was analysed. In that study, by defining overweight and obesity in children as functions of age and sex, Cole et al. developed specific cut-off percentiles for BMI by age and sex in children in the year 2000 [6]. These children must be on the BMI percentile curves that cross the values of 25 kg/m^2^ and 30 kg/m^2^ for overweight and obesity, respectively, at 18 [5-7].

There is a multifactorial interplay to the cause of childhood obesity ranging from genetic, environmental, socioeconomic, and cultural factors and other factors contributing to this weight or BMI changes. BMI does not directly measure body fat but correlates with other direct measures of body fat, such as dual-energy X-ray absorptiometry, underwater densitometer, bioelectrical impedance, and other methods [2,8,9]. In children, these body fats accumulate when energy inputs exceed energy outputs, and thus ultimately lead to weight gain. Children's most source of energy inputs is food, while energy output comprises physical activities and energy required for the body's metabolic activities and physiological processes. Also, in the absence of underlying disease, genetic inheritance may compound weight gain [2-6,8-12].

When the body takes in more calories than it uses up, obesity occurs. According to studies, an excess of 50-100 calories per day will accumulate about six to ten pounds over a year, causing little imbalance in energy balance to generate a significant weight gain over time. This imbalance in the caloric theory of obesity in children has been validated with studies that show that obese children did not necessarily consume more calories when adjusted for age, sex, ethnicity, socioeconomic and other factors among their peers [1,2,5-12].

Recent studies have begun to examine this hitherto "under-noticed" pandemic in detail and have uncovered its enormous public health ramifications regarding childhood illnesses, adult obesity, and future threats. An obese child is at increased risk of psychological and medical complications. These complications can be short-term or long-term [1,2,5-12]. Some previously thought complications of childhood obesity have now been shown to occur in the pediatric population [1,2,5-12]. These findings are concerning and have triggered several guidelines to be developed and continuously modified to mitigate this global epidemic before it consumes the generations to come [9-13]. The last two decades of the previous century have witnessed a dramatic increase in healthcare costs due to obesity and related issues among children and adolescents [14,15]. This study, therefore, aims to review the current obesity trends in the United States, the documented associated consequences of this menace, and attendant guidelines as they have evolved over the last 20 years.

## Materials and methods

We used the National (Nationwide) Inpatient Sample (NIS) database to conduct a retrospective analysis of patients with primary or secondary obesity from 2016 to 2019. We used the International Classification of Diseases (ICD)-10 diagnosis codes E66, E660, E6601, E6609, E662, E668, and E669 to extract the obese population. We excluded individuals with drug-related obesity or obesity due to a known pathologic condition unrelated to excessive calorie intake (see inclusion and exclusion criteria in Table 1 below). First, we queried the entire age population, then grouped them according to age category and selected our population of interest, i.e., age 18 and below. The NIS is a database that has been deidentified and is open to the public. It contains information on over 8 million hospitalizations each year, which accounts for approximately 20% of all admissions in the United States. Also, we conducted a literature search in PubMed and Google Scholar for updates on the guidelines regarding childhood obesity management using the MeSH terms "Childhood Obesity," "Obese," "Obesity," "Childhood Overweight," "Definitions," "Guidelines," "Management," and "Changes" using the variable linking terms of "AND," "OR," and "WITH" to search for relevant literature between 2000 and date.

**Table 1:** Inclusion and exclusion criteria NIS: The National (Nationwide) Inpatient Sample; ICD: International Classification of Diseases

Inclusion criteria	Exclusion criteria
NIS inpatient data on obesity using ICD codes E66, E660, E6601, E6609, E662, E668, and E669.	ICD codes that did not cover obesity-related conditions.
Pediatric population 18 years or less	All population over 18 years
Obesity due to calorie excess	Obesity due to medication or medical condition

Statistical design

We used a quantitative statistical approach to analyze our study's variables, including sex, age, ethnicity, median family income, and geographic areas (northeast, midwest, south, and west). We represented continuous variables as means with standard deviation and non-continuous variables as percentages. Results with p-values less than 0.05 were taken to be statistically significant. Version 9.4 of the Statistical Analysis System (SAS) software was used for all analyses (SAS Institute Inc., Cary, USA).

## Results

Result of database extraction by the total prevalence

Table 2 below shows the number of obese patients (both children and adults) hospitalized with an ICD 10 diagnosis code related to obesity between 2016 and 2019. There were 28,484,087 obesity-related between 2016 and 2019. Of these, 13.9% (3,946,889) of them were diagnosed with obesity.

**Table 2:** Cross-sectional prevalence of total obesity between 2016 and 2019

	Obesity-related admission	Proportion
Population without obesity	24,537,198	86.14%
Population with obesity	3,946,889	13.86%
Total admission	28,484,087	

​​​​​​Result of obesity prevalence by age-adjusted (18 years or younger) pediatric population

When age-adjusted, the sample population for those 18 years or under is represented in Table 3 below. We found that 62,669 (1.5%) were children with obesity with a mean age of 14 (SD = 4). Also, there was a 64.2% female preponderance. We obtained a yearly trend and found a steady and significant rise from 2016 to 2018 (24% vs. 26%), with a slight decline in 2019 (25%; p < 0.001).

**Table 3:** Age-adjusted (18 years or below) obesity prevalence between 2016 and 2019

	2016	2017	2018	2019	
Prevalence	14,786	16,004	16,320	15,559	62,669
Cumulative percentage	23.59%	25.54%	26.04%	24.83%	100% (p<0.0001)

Sex-adjusted rate of childhood obesity

Table 4 below contains the crude sex-adjusted rate.

**Table 4:** Sex-adjusted prevalence of childhood obesity between 2016 and 2019

Male	Female	Total
22,460 (35.85%)	40,196 (64.15%)	62,656 (100%)

Race-adjusted childhood obesity prevalence

Tables 5 and 6 below summarizes the pooled race-adjusted prevalence. Even though the white population had the highest overall prevalence of childhood obesity (40.9%), the Native Americans, Hispanics, and black population had a higher prevalence per ethnicity, with a 0.5% and 0.33% prevalence, respectively, compared to 0.14% in the white population (p < 0.0001).

**Table 5:** Race-adjusted childhood obesity between 2016 and 2019

	Whites	African Americans	Hispanics	Asian or Pacific Islanders	Native Americans	Others	
Prevalence	24,310	13,590	17,007	1,124	752	2,732	5,9515
Percentage	40.85%	22.83%	28.58%	1.89%	1.26%	4.59%	100 (p < 0.0001)

**Table 6:** Ethnicity adjusted per total admission within the ethnic group

Pediatric obesity	Race/Ethnicity						
	Whites	Blacks	Hispanics	Asians	Native Americans	Others	Total
Yes	24,310	13,590	17,007	1,124	752	2,732	≈ 62669; p < 0.0001
Prevalence per ethnicity	0.14%	0.33%	0.50%	0.13%	0.41%	0.28%	1.5%; p < 0.0001
Total prevalence	64.95	15.27	12.49	3.11	0.67	3.51	100.00%; P < 0.0001

Regional obesity trends

Table 7 below shows the trends in the various geographical regions when we adjusted by region.

**Table 7:** Region-adjusted childhood obesity prevalence between 2016 and 2019

	Northeast	Midwest	South	West	
Prevalence	9,610	14,767	22,809	15,483	62,669
Percentage	15.33%	23.56%	36.40%	24.71%	100% (p < 0.0001)

Childhood obesity by household income

Table 8 below summarizes our findings when adjusted by household income. 

**Table 8:** Median household income national (quartile for patient zip code) vs. childhood obesity prevalence

	0-25th percentile	26-50th percentile	51-75th percentile	76-100th percentile	
Prevalence	23,617	16,361	13,735	8,163	61,876
	38.17%	26.44%	22.20%	13.19%	100% (p < 0.0001)

## Discussion

**Figure 1:**
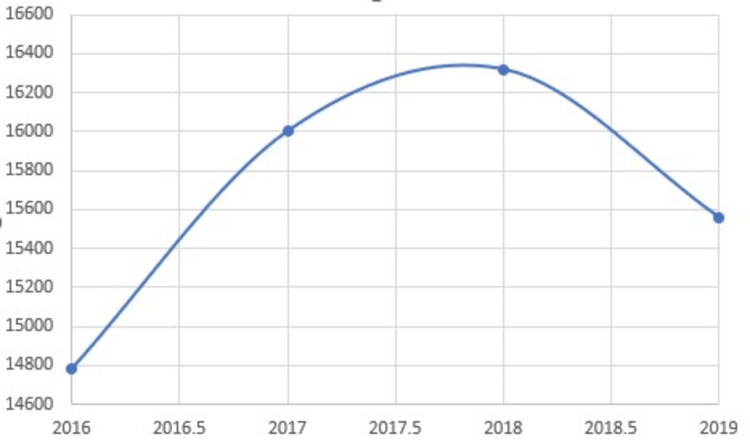
Crude prevalence between 2016 to 2019

The rate of obesity has remained on the rise over the last decade. In our analysis (Figure 1), there was a 13.86%-point prevalence between 2016 and 2019 and a cumulative increase from 23.59% (14,786) in 2016 to 26.04% (16,320) in 2018 but a slight decline to 24.83% (15,559) in 2019. This cumulative increasing trend is similar to previous years, which was reported at 11.6% in a sample between 2005-2015 [1,2,6-12,16-18]. 

In determining the prevalence of childhood obesity in children aged 18 and under, we discovered that 62,669 (1.5% of the sample) were obese and had a mean age of 14 (SD = 4). When evaluating the trend and point prevalence for each year between 2016 and 2019, we found that there was a continuous increase that plateaued between 2017.5 and 2018, with 2019 recording a slight decrease. These increasing inpatient statistics are consistent with the general population's age-adjusted rates, which increased from 30.5% in the early 2000s to around 42.4% in 2018 [17,18]. 

In terms of sex-based differences, it has been known that obesity is more prevalent amongst male children globally, but this is contrary to our findings [18,19]. Our data showed a preponderance of female obese children (64.2%) than males between 2016 to 2019. Similar other studies and reports [1-6,8-20] from the general population in the adult population have also shown this higher trend of obesity in females than in males. Several studies have attributed the preponderance of obesity in male children over females to several factors. These observed disparities have been linked to various biological causes, including a larger fat mass at birth, which is associated with less energy and lower caloric demands in females than boys. Furthermore, a higher androgen level in males has a suppressive effect on leptin. Leptin, a hormone that inhibits hunger and enhances energy consumption, has been discovered to be higher in female children than in their male counterparts [19-25]. This greater male-to-female obesity prevalence in children contradicts the female adult population's predominance of obesity over men, ascribed to a more significant amount of brown adipose tissues [19-22].

Ethnicity also plays a massive role in how obesity is skewed. Our analysis showered a statistically significant higher rate in people of color. Even though the white population had the highest overall prevalence of childhood obesity (40.9%), the Hispanic and black populations had a higher prevalence per population, with a 0.5% and 0.33% prevalence, respectively, compared to 0.14% in the white population (p < 0.0001). Attempts to explain the greater obesity prevalence in lower-income groups argue that kids from low-income individuals are likely to be obese, overweight, and have poor health in general because they are more concentrated in environments that do not support physical activities and healthy eating [23].

Furthermore, in our study, data from the southern geographical region showed more preponderance of obesity compared to the other areas. This disproportionately higher obesity in the southern states has been similarly reported in several other studies [23,24]. Also, in keeping with previous trends of higher obesity in lower-income families, our analysis showed that the higher the household income, the lesser the childhood obesity rate. 

**Figure 2:**
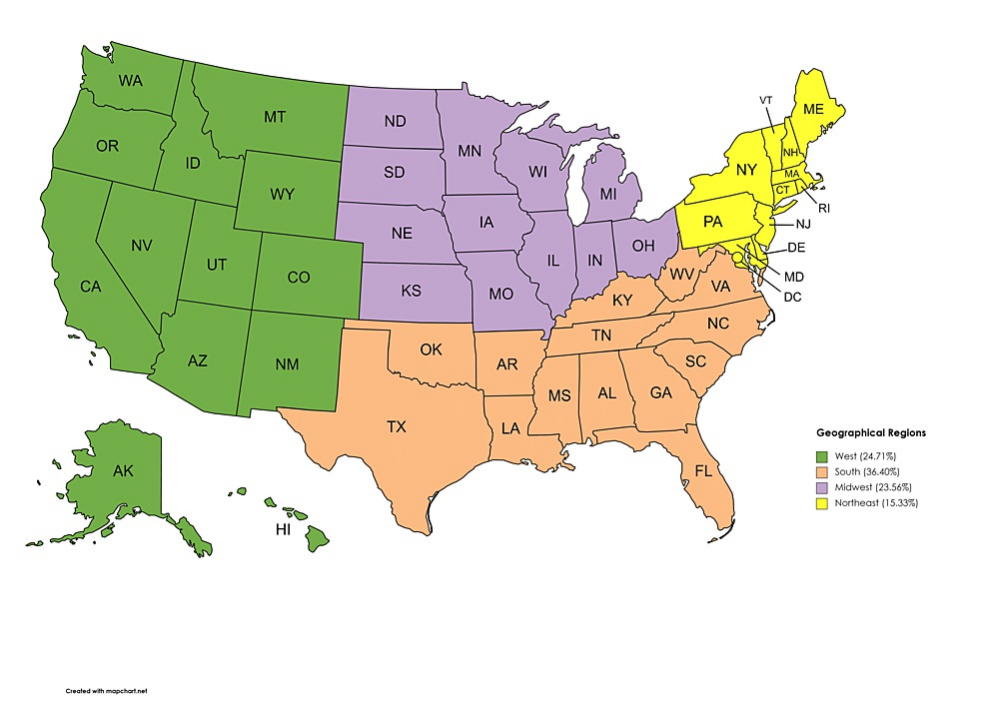
Prevalence of childhood obesity by geographical region This map was originally created by the authors.

Complications and recommendations

This rising prevalence has both health and economic implications. First, from a financial point of view, the rise is directly linked to the increasing cost of healthcare for this group of people. In a pooled cross-sectional analysis between 2001 and 2016 to assess the direct cost of medical care at the state and national levels in the United States, Cowey et al. reported that adults with obesity incurred a 100% ($2,505) increase in medical cost at an individual level, with a significant cost increase from 68.4% to 233.6% from Class 1 to Class 3 obesity, respectively [14]. This increasing prevalence of obesity raised costs in every care category, like sub-forms of outpatient and inpatient care [14,26].

Obesity in childhood has been related to a variety of disorders. It is also a predictor of adult obesity, which may lead to obesity-related disorders if not well controlled later in life. High blood pressure, type 2 diabetes, asthma, obstructive sleep apnea, heart disease, obesity-induced hypoventilation, many orthopedic and surgical problems, and even malignancy are among these conditions [19-27]. More recently, research by the American Institute for Cancer Research published in November 2009 revealed that obesity is connected to more than 37,000 new cancer cases per year [27]. In line with these obesity-linked morbidities, several guidelines have been developed to mitigate the effect of the scourge of obesity in the pediatric population [24-27].

The American Psychology Association's recommendations

**Figure 3:**
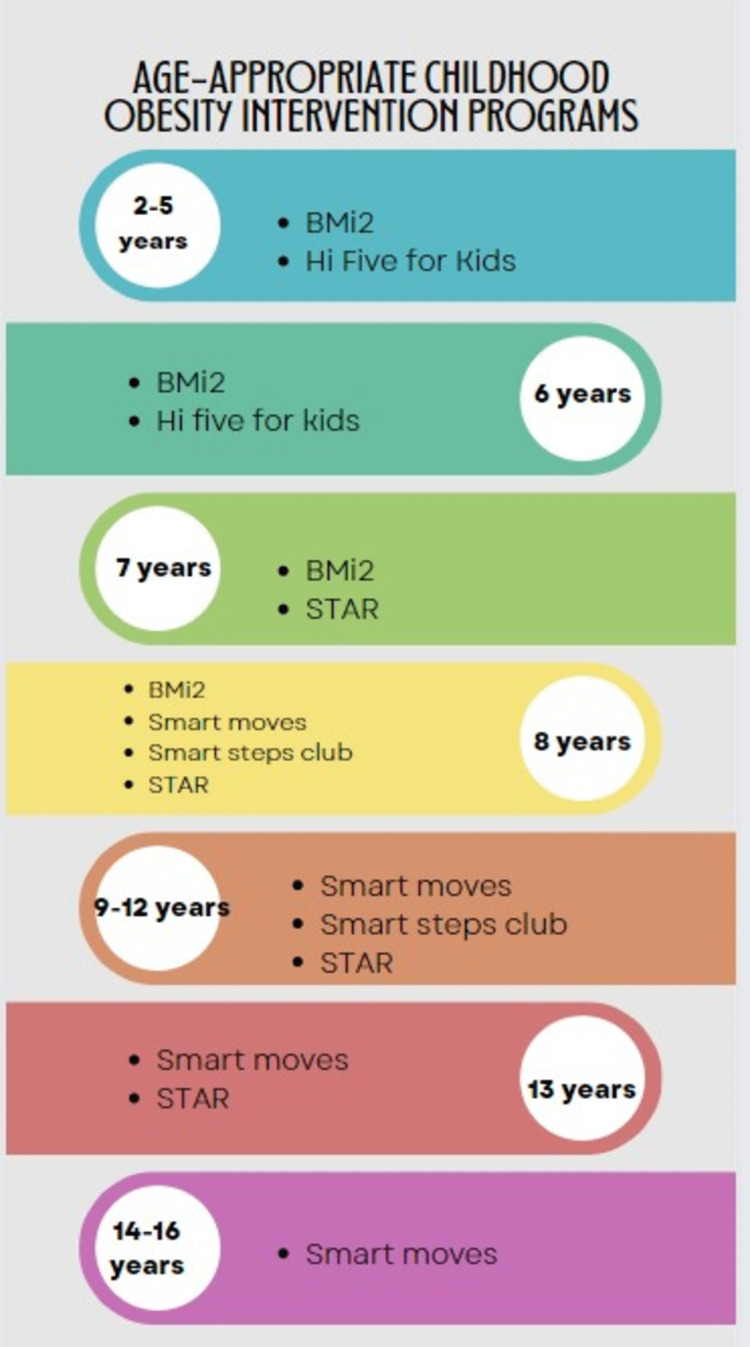
Age-appropriate childhood obesity intervention programs 1. BMi^2^: Created by the School of Public Health at the University of Michigan [25] 2. High five for kids: developed by Harvard Pilgrim Health Care Institute [25] 3. Smart Moves™: created by the Yale School of Medicine's Bright Bodies Weight Management Program for Kids [25] 4. Smart Steps Club: Created by the Children's Hospital of Philadelphia [25] 5. STAR intervention: Designed by the Harvard Pilgrim Health Care Institute [25] BMi^2^: Brief Motivational Interviewing to Reduce Body Mass Index; STAR: Study of Technology to Accelerate Research

Overall, childhood obesity management recommendations have continued to stress prevention and therapy where prevention is not practicable. The American Psychology Association's 2018 clinical recommendation for treating childhood and adolescent obesity suggests integrating food, behavioral modification, and physical exercise of sufficient intensity to improve children's and adolescents' overall health and development. Their recommendation is a family-based multi-component strategy in which the focus is not just on the child's weight but also on the child's general health, considering features such as age, gender, family culture, and aptitude. Their suggestion includes using many age-specific intervention programs to be administered via a multispecialty approach, including age-specific behavioral and dietary adjustments. Dietitians, for example, focus on assisting families in making the right beverage and diet choices, psychologists focus on the barriers to physical and behavioral change, physicians monitor the overall health, and exercise experts may assist families in incorporating fun exercises to help them lose weight and include daily physical activities in the lives of their children. The Brief Motivational Interviewing to Reduce Body Mass Index (BMi^2^), high five for kids, Smart Moves^TM^, Smart Steps Club, and STAR (Study of Technology to Accelerate Research) intervention programs are age-appropriate weight loss programs designed by various health or academic institutions for children and adolescents that include different degrees and types of diet, physical activities, and behavioral modification patterns [25]. Figure 3 below shows a summary of these multicomponent, multidisciplinary intervention recommendations.

The CDC's recommendations

Similarly, the US Preventive Services Task Force recommends early evaluation of young children and adolescents for obesity and referring or offering those who are obese comprehensive family-centered, intensive behavioral therapies to achieve weight loss (CDC3). As part of the holistic intervention strategy, they recommend screening tools such as clinical growth charts and BMI calculators, as well as adopting other best practice evidence-based guidelines from the American Academy of Pediatrics and National Academy of Medicine frameworks and referring to community resources.

The American Academy of Family Physicians's recommendations

The American Academy of Physicians recommends early screening for overweight and obesity [2-9]. It encourages primary care physicians to identify risk factors and other socioeconomic factors that promote the development of obesity, such as low physical activity and unhealthy feeding habits. When these are identified, prompt management approaches should be triggered, such as providing fast weight control programs and other obesity intervention programs, providing patient-centered counseling, using motivational interviewing techniques, and referrals to intervention programs like the Special Supplemental Nutrition Program for Women, Infants, and Children (WIC) for those in the socioeconomic risk category [2-9]. 

The guidelines for therapy include four phases of obesity care: first is a short counseling session conducted in a healthcare office, and the succeeding steps demand more resources and time. The appropriateness of higher phases is determined by the degree of overweight or obesity and the patient's age. These recommendations acknowledge the significance of an environmental and social change in combating the obesity epidemic. Still, they outline how healthcare practitioners and institutions may contribute to more significant initiatives.

They also created a comprehensive childhood obesity prevention policy, including resources for healthcare providers that use a tiered approach (i.e., federal, state, school, community, and practice levels) [26]. This policy tool outlines specific strategies for fostering healthier environments. These policy tools incorporate adopting early screening tools for obesity, enabling a breastfeeding environment, limiting access to unhealthy foods, and increasing access to healthy foods and the point of purchase. They also include restricting screen time to about two hours, increasing physical activities and access to safe and attractive places for physical activity, limiting access to unhealthy beverages, creating access to healthy drinks, and promoting holistic media campaigns [26]. 

Even though these intervention programs and nutritional policies have been reviewed, there are still gaps in the impact of food availability and regulations on childhood obesity reduction, as most of the studies used to develop these interventions had varying degrees of methodological flaws. These flaws include selective bias, as the literature search shows that most of the studies on which these recommendations are based were done in school intervention programs in high-income societies. However, more research in resource-poor settings may be required, or the analysis may need to be stratified to include subgroups such as age, gender, socioeconomic status, and race/ethnicity. In addition, micro-level research may help identify specific response rates to the same intervention, maximizing future benefits or interventions.

Though the US Food and Drug Administration has approved several modern medications for usage in youngsters, such as phentermine in children over the age of 12 for weight reduction, the weight reduction is minimal to moderate [9,28]. Tremors, anxiety, and slightly elevated blood pressure are all possible side effects of phentermine. In addition, topiramate-extended release, which has typically been used as an antiepileptic, can also help decrease cravings, but the mechanism is unknown; it is also FDA-approved [6-14,28]. Furthermore, for kids with greater than the 95th percentile weight, adjusted for age and sex (BMI >35 kg/m^2^ with severe to moderate comorbidities or BMI >40 kg/m^2^), bariatric surgery procedures are on the increase and are reserved as a therapy for morbid obesity in adolescents, especially when skeletal and sexual maturity are affected. Structured weight management programs like Jenny Craig Weight Loss Centers and Weight Watchers will take older children with parental and medical approval. The Shapedown Pediatric Obesity Program is an organized weight loss and weight control program for children aged six to 20. It has offices in all 50 states across the country [5-9].

Prevention

Efforts to develop obesity prevention strategies for normal-weight children are ongoing, but these recommendations have weaknesses in randomized controlled trials and evidence. However, they may suffice as the evidence keeps evolving. The CDC and other health authorities in the United States recommend several approaches: active participation from all parties, especially primary care offices, in using the proper screening tools when they see these kids and their families for medical care, evaluation of the kids' diets and physical activities as a whole, and quick referral to full multidisciplinary intervention and programs when obesity is diagnosed; improving healthy eating habits (lean proteins, fat-free or low-fat diets, whole grains, and lots of vegetables); improving and increasing time for physical activities for the pediatric population, to at least 60 minutes for children aged six to 17; a family-based physical activity approach by involving them in family activities as a family-based approach; reducing screen time and improving their sleep time (eight to ten hours for teenagers 13 to 18 years, nine to 12 hours for kids six to 12 years, and for preschoolers to have about 11 to 13 hours of sleep); further recommendations [18,26,27,29-31].

Study limitation

This study was based on the NIS database, which represents about 20% of the national admission in the United States. Also, the weighted data were compounded by missing or incomplete entries that were accounted for with variance during statistical calculations using the "listwise deletion technique" or complete case analysis by omitting the missing data and analyzing the remaining complete data. 

## Conclusions

In our finding, obesity in the pediatric population is still increasing, continuing on its previously recorded trajectory. Various recommendations from health policymakers have bolstered efforts to tackle this escalating pandemic. However, additional information on the compliance, use, and adherence to these policies by healthcare professionals and members of the public, as well as the consequence of utilization or compliance to these guidelines, is needed. Nevertheless, given the continuous growth of childhood obesity, despite the avalanche of these recommendations, the issue of compliance arises, or other essential risk factors might have been overlooked. Additional studies may be needed to unmask this looming phenomenon.

## Appendices

The authors performed the following roles: 

1.       Okelue Edwards Okobi: Conceptualization, data curation, formal analysis and interpretation of data, investigation, methodology, project administration, resources, software, validation, visualization, writing - original draft, writing - review and editing, supervision, oversight, and leadership.

2.        Ijeoma C. Izundu: Conceptualization, review, investigation, methodology, and project administration.

3.       Endurance O. Evbayekha: Resources, validation, visualization, writing - original draft, and data analysis.

4.       Emmanuel Egberuare: Project administration, resources, validation, visualization, and writing - review and editing.

5.       Esther Segun: Data curation, formal analysis, review and editing, and cross-referencing.

6.       Rufiat Abdulgaffar: Article plan and commitment (APC) drive, fact-checking, project administration, resources, oversight, and leadership.

7.        Tunde Oyelade: Validation, visualization, writing - review and editing, and leadership.

8.       Aisha M Abubakar: Conceptualization, validation, visualization, and review - second draft,

9.       Jenny Jackson Onyema: Visualization, writing - fact-checking of the original draft, resources, and validation.

10.     Tariladei S. Peresuodei: Visualization, edits, fact-checking, and idealization.

11.      Scholastica Uyileubenye Abu-Undiyaundeye: Ideation, visualization, and preliminary data verification.
